# Circulating extracellular vesicles as novel biomarkers for pulmonary arterial hypertension in patients with systemic lupus erythematosus

**DOI:** 10.3389/fimmu.2024.1374100

**Published:** 2024-09-19

**Authors:** Zhe Ding, Fumin Qi, Li Liu, Zhouming Wang, Na Zhang, Xing Lyu, Wenwen Sun, Jun Du, Haoming Song, Hou Hou, Ying Guo, Xiaomei Wang, Ming-Lin Liu, Wei Wei

**Affiliations:** ^1^ Department of Rheumatology and Immunology, Tianjin Medical University General Hospital, Tianjin, China; ^2^ Tianjin Clinical Research Center for Rheumatic and Immune Diseases, Tianjin Science and Technology Bureau, Tianjin, China; ^3^ Department of Neurosurgery, Tianjin Institute of Neurology, Tianjin Medical University General Hospital, Tianjin, China; ^4^ Department of Cardiovascular, Tianjin Medical University General Hospital, Tianjin, China; ^5^ Department of Cardiology, Tongji Hospital, Tongji University School of Medicine, Shanghai, China; ^6^ Corporal Michael J. Crescenz Veterans Affairs Medical Center (VAMC), Philadelphia, PA, United States; ^7^ Department of Dermatology, Perelman School of Medicine, University of Pennsylvania, Philadelphia, PA, United States

**Keywords:** extracellular vesicles, systemic lupus erythematosus, pulmonary arterial hypertension, biomarker, procoagulant

## Abstract

**Introduction:**

Pulmonary arterial hypertension (PAH) is a serious complication of systemic lupus erythematosus (SLE) with increased mortality. A prothrombotic state may contribute to pathogenesis of SLE-PAH. Extracellular vesicles (EVs) are known to be associated with thrombosis. Here, we investigated circulating EVs and their associations with SLE-PAH.

**Methods:**

Eighteen SLE-PAH patients, 36 SLE-non-PAH patients, and 36 healthy controls (HCs) were enrolled. Flow cytometry was used to analyze circulating EVs from leukocytes (LEVs), red blood cells (REVs), platelets (PEVs), endothelial cells (EEVs), and Annexin V^+^ EVs with membrane phosphatidylserine (PS) exposure.

**Results:**

Plasma levels of all EV subgroups were elevated in SLE patients with or without PAH compared to HCs. Furthermore, plasma Annexin V^+^ EVs, LEVs, PEVs, REVs, EEVs, and Annexin V^+^ REVs were significantly elevated in SLE-PAH patients compared to SLE-non-PAH patients. Additionally, PAH patients with moderate/high SLE showed a significant increase in LEVs, PEVs, REVs, Annexin V^+^ EVs, and Annexin V^+^ REVs compared to SLE-non-PAH patients. However, PAH patients with inactive/mild SLE only exhibited elevations in Annexin V^+^ EVs, REVs, and Annexin V^+^ REVs. In the SLE-PAH patients, EEVs were positively correlated with pulmonary arterial systolic pressure, while PEVs and EEVs were positively correlated with right ventricular diameter. Moreover, the receiver operating characteristic curve indicated that Annexin V^+^ EVs, LEVs, PEVs, REVs, EEVs and Annexin V^+^ REVs could predict the presence of PAH in SLE patients. Importantly, multivariate logistic regression analysis showed that circulating levels of LEVs or REVs, anti-nRNP antibody, and serositis were independent risk factors for PAH in SLE patients.

**Discussion:**

Findings reveal that specific subgroups of circulating EVs contribute to the hypercoagulation state and the severity of SLE-PAH. Higher plasma levels of LEVs or REVs may serve as biomarkers for SLE-PAH.

## 1 Introduction

Systemic lupus erythematosus (SLE) is a complex autoimmune disease affecting multiple organs with various clinical manifestations. Pulmonary arterial hypertension (PAH) is a serious complication of SLE, leading to death due to progressive right heart failure (1). PAH is characterized by increased pulmonary vascular resistance caused by different etiologies and pathogeneses (1, 2). Connective tissue diseases (CTDs) are the important cause of PAH. Systemic sclerosis (SSc)-associated PAH (SSc-PAH) is the most common type of CTD-PAH in Caucasians (2). However, SLE is the most common CTD-associated PAH in Chinese (2). Lack of specific clinical symptoms makes the early diagnosis of PAH difficult. PAH is an independent prognostic factor of SLE, as SLE-PAH patients experience significantly worse clinical outcomes than SLE-non-PAH patients (3). Without appropriate treatment, SLE-PAH patients face a tragically short average survival time of only two years from the onset of PAH (4). Thus, improving the prognosis of SLE-PAH patients remains as a critical challenge for rheumatologist. Although the pathogenesis of SLE-associated PAH still remains unclear, emerging evidence suggests the potential involvement of hypercoagulation and thrombosis in its development (5). However, the unclear risk-benefit profile of anticoagulantion therapy in SLE-PAH patients without antiphospholipid antibodies poses a challenge for treatment decisions (6–8). Therefore, better understanding of coagulation status in SLE-PAH patients may provide valuable insights into diseases prognosis and pave the way for novel therapeutic strategies.

Extracellular vesicles (EVs) are cellular membrane structures that are released from cells when they are stimulated or undergo different types of programmed cell death (9–11). Studies from our and others have demonstrated that EVs may harbor proteins, lipids, nucleic acids, or multi-molecular complexes from the nucleus, cytoplasm, or plasma membrane of their parental cells (10, 12). Functionally, EVs can serve as novel mediators for intercellular communication within organs or across different organ systems (10). Among the diverse array of biological features EVs possess, their roles in procoagulation remain the most studied function, with implications for various human diseases. Our previous studies have shown that EVs from monocyte/macrophages, with cholesterol enrichment or stimulation by tobacco smoke extracts, have potent procoagulant activities due to their-associated phosphatidylserine (PS) and other procoagulant factors (13, 14). EV shedding process results in exposure of membrane PS, a phospholipid crucial for procoagulant activity by providing surface for assembly of prothrombin complex, thus promoting thrombus formation (13, 14). Annexin V, a calcium-dependent phospholipid-binding protein that can preferentially bind PS on EV surface, becomes a valuable tool for detecting EVs with procoagulant functions (13, 14). In addition, circulating EVs derived from platelets (PEVs), red blood cells (REVs), leukocytes (LEVs), and endothelial cells (EEVs) (15) are also important in cardio-pulmonary diseases (16, 17).

Various studies from our and other teams have shown that EVs contribute to PAH, with elevated procoagulant conditions or even pulmonary embolism (18, 19). However, circulating EVs and their pathogenic involvement in PAH associated with SLE remain unexplored. Furthermore, the inconsistent survival advantage of anticoagulant therapy for SLE-PAH patients compared to idiopathic pulmonary arterial hypertension (iPAH) and SSc-PAH (6–8) suggests that the underlying mechanisms of SLE-PAH may not be fully captured by previous studies. In the current study, we systematically analyzed the levels of circulating EVs from different cell types, such as LEVs, REVs, PEVs, or EEVs, and procoagulant Annexin V^+^ EVs, using flow cytometry, to determine the potential involvement of circulating EVs in pathogenesis of PAH in SLE patients. Through this study, we sought to provide evidence for the involvement of circulating EVs in development of PAH in patients with SLE, paving the way for new therapeutic targets or diagnostic tools.

## 2 Materials and methods

### 2.1 Study population and data collection

Subjects were enrolled from August 2019 to December 2021 at the Department of Rheumatology in Tianjin Medical University General Hospital. A total of 18 SLE-PAH patients; 36 SLE-non-PAH patients with matched age, sex, and SLE disease activity (SLEDAI-2K) (20); and 36 healthy volunteers with matched age and sex were recruited to the study. All 54 SLE patients met the 2019 European League Against Rheumatism/American College of Rheumatology classification criteria for systemic lupus erythematosus (21). Seven PAH patients were diagnosed by right heart catheterization (RHC) with criteria of a mean pulmonary arterial pressure (mPAP) ≥ 25 mmHg at rest, a pulmonary capillary wedge pressure (PCWP) ≤ 15 mmHg, and pulmonary vascular resistance (PVR) > 3 Wood Units (22); while the other 11 PAH patients were diagnosed by pulmonary arterial systolic pressure (PASP) > 50 mmHg with ultrasonic cardiogram (UCG). Other causes of PAH (23) were excluded to ensure that PAH was attributable to SLE. Although rare, SLE patients can have interstitial lung disease (ILD). When forced vital capacity was less than 60% predicted or, fibrosis lesion was large than 1/3 lung fields on high-resolution computed tomography (HRCT), the patients were also excluded to avoid ILD-associated PAH (2). PASP, as estimated by tricuspid regurgitation in UCG of SLE-non-PAH patients, could not be more than 30 mmHg. This study was approved by the Institutional Medical Ethics Review Boards of Tianjin Medical University General Hospital and informed consent was obtained from all study participants.

The following clinical data were collected from medical records: demographics, medical history, symptoms and syndromes, RHC results, laboratory tests (routine examinations of blood, urine and stool, phase microscope for hematuria, liver and renal function, coagulation function, indexes correlated with immune status, inflammatory markers, 24 hours proteinuria), and imageological examination (UCG, abdominal ultrasound, pulmonary HRCT, and magnetic resonance imaging of brain).

### 2.2 Sample preparation

All study subjects fasted for more than 8 h before venous blood samples were collected between 7 a.m. and 9 a.m. Three milliliters of peripheral blood was collected in a Vacutainer™ (BD Biosciences, San Jose, CA, USA) containing 3.2% sodium citrate (volume of anticoagulation: volume of blood = 1:9) and processed within 2 h. The sample was centrifuged at 120 x *g* for 20 min to obtain the platelet-rich plasma supernatant. This was then centrifuged at 1,500 x *g* for 20 min and the platelet-poor plasma supernatant was collected. The platelet-poor plasma was further centrifuged at 13,000 x *g* for 3 min to obtain cell-free plasma (CFP). All centrifugations were performed at 20°C and no more than one freeze-thaw cycle was permitted.

### 2.3 Flow cytometry analysis

**Figure 1:**
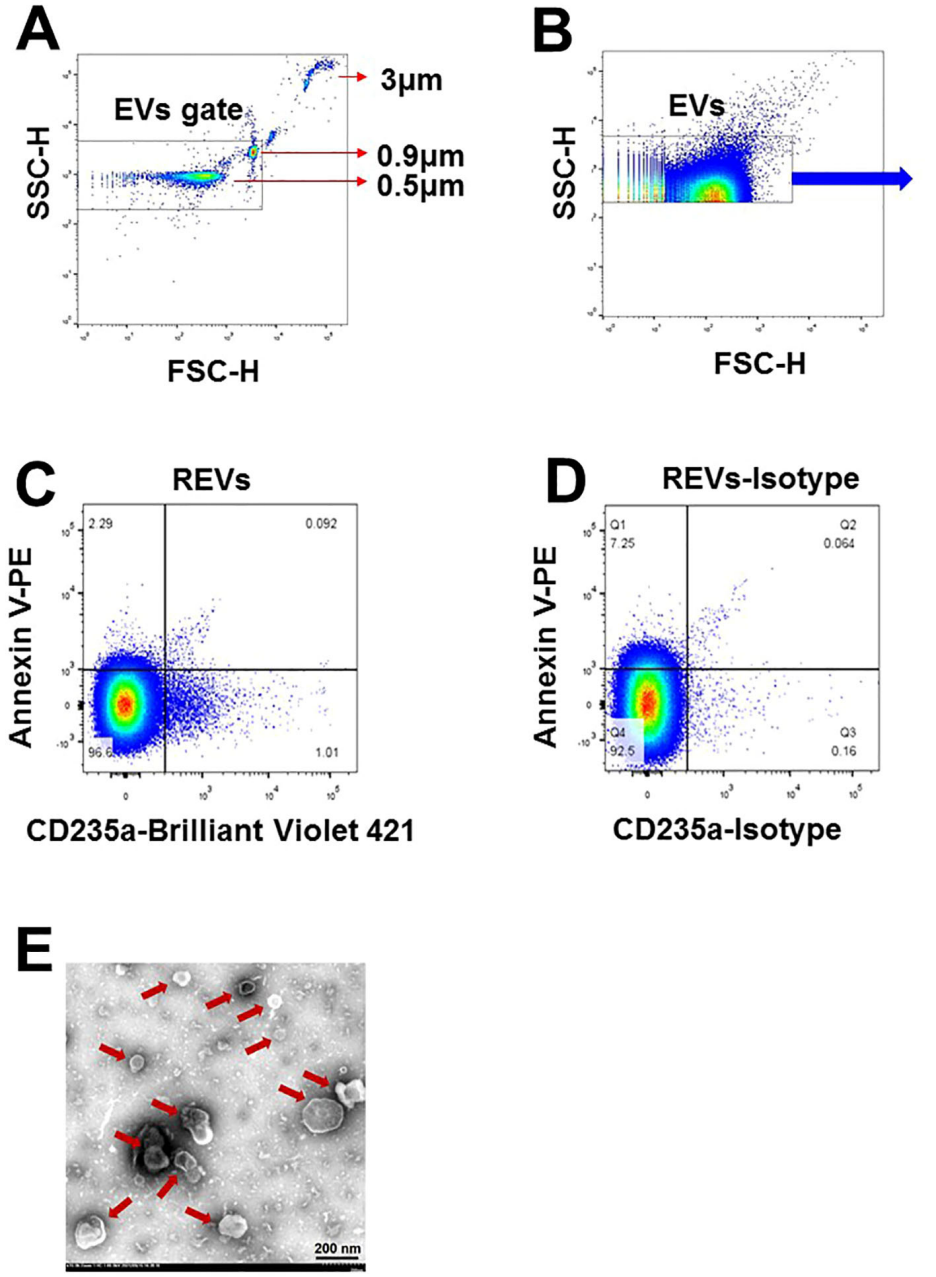
Characterization of EVs in plasma of SLE-PAH patients. **(A)** Microbeads of 0.5, 0.9, and 3µm in diameter were used for gating EVs in flow cytometry analysis. **(B)** EVs were gates for further analysis. **(C, D)** Flow cytometry of EVs and their isotypes: erythrocytes-derived EVs. **(E)** Transmission electron microscopy images of isolated EVs of varying size from the plasma of SLE patients. Arrows indicated individual EVs of different size.

A panel of cell-specific monoclonal antibodies was used to label EVs originating from leukocytes (CD45), erythrocytes (CD235a), platelets (CD41a), and endothelial cells (CD144). Among the fluorescent antibody reagents, CD45-Brilliant Violet 650 (BV650) (BD Biosciences Cat# 563717, RRID : AB_2738387), CD41a-fluorescein isothiocyanate (FITC) (BD Biosciences Cat# 555466, RRID : AB_395858), CD235a-Brilliant Violet 421 (BV421) (BD Biosciences Cat# 562938, RRID : AB_2721016), CD144-Alexa Fluor 647 (BD Biosciences Cat# 561567, RRID : AB_10712766), and their isotype-matched control conjugates ((BD Biosciences Cat# 563231, RRID : AB_2869470), (BD Biosciences Cat# 555748, RRID : AB_396090), (BD Biosciences Cat# 562748, RRID : AB_2721018), and (BD Biosciences Cat# 557714, RRID : AB_396823)) were procured from BD Biosciences (San Jose, CA, USA). Annexin V–phycoerythrin (PE) was obtained from BioLegend (San Diego, CA, USA) (BioLegend Cat# 640908, RRID : AB_2561298), and the negative control abolished the interaction of Annexin V with PS by the addition of ethylene diamine tetraacetic acid. The number of EVs were determined using high-sensitivity flow cytometry (BD LSRFortessa™ Cell Analyzer, BD Biosciences) following a previously described standardization method. Briefly, the upper limit of the EVs quadrant gate was established at 1 μm using standard microbeads that measured 0.5, 0.9, and 3 µm in diameter. The lower limit was determined with a threshold at side-scatter 200 nm (**Figures 1A, B**). Fifty microliters of CFP was incubated with the appropriate amount of antibodies of the parental cell type for 30 min. The mixtures were then incubated with 5 μL Annexin V and 50 μL 2× Annexin V binding buffer (BD Biosciences) for 15 min. All incubations were performed at 20°C in the dark. After incubation, 10 μL count beads (Spherotech, Lake Forest, IL, USA) were added to the sample to calculate the absolute concentration of EVs, and all the samples were diluted to 500 μL with phosphate buffered saline (PBS) before analysis with flow cytometry (**Figures 1C, D**). The absolute EV counts were determined using the following equation: (A/B) × (C/D) = number of EVs per microliter. In this computational formula, A was the number of events for the test sample, B was the number of events for the count fluorescent beads, C was the total number of count fluorescent beads per 10 μL, and D was the volume of test sample initially used. All the buffers used in the EVs detection were filtered with 0.1 μm filters (Sartorius, Göttingen, Germany) to avoid contamination from non-cellular particles. Prior to use, the inner and outer tubes of the cytometer were completely cleaned until the background detection was fewer than 80 events/s. Sample analysis was run at a low speed and collection stopped at 60s.

### 2.4 Transmission electron microscopy

Six milliliters of fresh CFP was centrifuged at 100,000 x *g* for 1 h at 4°C to obtain the EVs sediment, which was then suspended in 60 μl PBS buffer to acquire pure EVs. A 150 Mesh carbon membrane/formvar membrane-coated grid was placed on a 30 μl EVs sample drop for 2 min at 20°C, and a filter paper was used to remove excess liquid. The sample was negatively stained by placing the grid on a 30 μl drop of phosphotungstic acid (pH 6.5) for 2 min followed by blotting and air drying. Digital images were obtained using a Hitachi HT-7700 transmission electron microscope (Tokyo, Japan). As shown in **Figure 1E**, the EVs were separate from each other and had a spherical-shaped double membrane structure.

### 2.5 Statistical analysis

The data with normal distribution were described as mean ± standard deviation (SD), and the median values were used to describe data with no normal distribution. The independent sample t-test was used to compare data for two groups, and the Mann-Whitney U-test was used if the data were not normally distributed. One-way analysis of variance, or the Kruskal-Wallis test for measurement data of abnormal distribution or unequal variances, was used to determine whether the variables in the three groups had significant differences.

Receiver operating characteristic (ROC) curves were used to detect the capacity of circulating EVs subgroups to discriminate SLE-PAH patients from SLE-non-PAH patients. Conditional logistic regression analysis was used to explore the risk factors for the PAH complication in SLE patients. Spearman’s rank correlation coefficient was used to examine the strength of the relationship between the EVs subgroups and the variables measured by UCG. All tests were two-sided, and *p* < 0.05 was considered statistically significant. Statistical analyses were performed using SPSS 25.0 for Windows (SPSS Inc., IBM Corp., Armonk, NY, USA).

## 3 Results

### 3.1 Demographics and baseline characteristics of all participants

In this study, total of 18 SLE-PAH patients (one male) were enrolled, including 4 patients with inactive SLE (SLEDAI-2K<4), 4 patients with mild SLE (5≤SLEDAI-2K<10), 3 patients with moderate SLE (10≤SLEDAI-2K<15), and 7 patients with severe SLE (SLEDAI-2K>15). In addition, 36 SLE non-PAH patients matched with age, sex, and SLE activity, and 36 healthy controls (HCs) matched with age and sex, were included. Whole blood cell parameters showed that red blood cells (RBC), hemoglobin (HGB), platelet (PLT), and hematocrit (HCT) levels were decreased in SLE patients with or without PAH as compared with HCs, while the indexes of RBC distribution width (RDW), coefficient of variation of RDW (RDW-CV), and standard deviation of RDW (RDV-SD) were increased (**Table 1**). Compared with SLE-non-PAH patients, RDW-CV and RDV-SD, uric acid level, and anti-nRNP antibody positive rate were higher in SLE-PAH patients. Analysis of coagulation function showed increased fibrinogen levels, prothrombin time (PT), and PT-international normalized ratio (PT-INR) in SLE-PAH patients who received a higher rate of anticoagulant treatment. Among the clinical manifestations of SLE, patients with SLE-PAH were more likely to have serositis and Raynaud’s phenomenon. UCG indicated that SLE-PAH patients had higher pulmonary artery systolic pressure (PASP), right ventricular diameter (RVD), and proportion of pericardial effusion, which were consistent with the patients’ condition. The other parameters were comparable between the SLE with and without PAH groups. The detail information about clinical and laboratory characteristics are shown in **Table 2**.

**Table 1:** Demographics and hematology analysis in SLE patients and healthy controls.

Variables	Healthy controls (n=36)	SLE-non-PAH (n=36)	SLE-PAH (n=18)	*p*-value	*p*-value for two groups		
					HC vs. SLE-non-PAH	HC vs. SLE-PAH	SLE-non-PAH vs. SLE-PAH
Demographics							
Age (years)	46.3±13.2	46.6±15.9	47.4±15.8	0.949	–	–	–
Gender (F/M)	34/2	34/2	17/1	1.000	–	–	–
Whole blood cell parameters							
WBC, ×10^9^/L	5.6 (5.1, 6.9)	5.2 (3.7, 6.7)	6.4 (4.1, 11.2)	0.116	–	–	
RBC, ×10^12^/L	5.0 (4.7, 5.4)	3.6 (3.1, 4.0)	3.7 (3.2, 4.3)	**<0.001**	**<0.001**	**<0.001**	0.732
HGB, g/L	137 (133, 141)	111 (90, 117)	99 (93, 128)	**<0.001**	**<0.001**	**<0.001**	0.811
PLT, ×10^9^/L	256 (211, 296)	202 (136, 259)	188 (100, 294)	**0.001**	**0.002**	**0.012**	0.666
HCT, %	42.7 (41.5, 43.6)	32.4 (28.1, 36.2)	32.8 (29.4, 39.8)	**<0.001**	**<0.001**	**<0.001**	0.388
RDW-CV, %	12.8 (12.2, 13.6)	13.8 (12.8, 16.0)	15.8 (14.3, 18.5)	**<0.001**	**0.002**	**<0.001**	**0.012**
RDW-SD, fL	40.9 (39.7, 42.9)	44.1 (41.3, 50.7)	53.3 (47.4, 57.7)	**<0.001**	**0.002**	**<0.001**	**<0.001**
PDW, fL	12.8 (1170, 13.8)	11.8 (10.6, 13.1)	10.7 (9.0, 16.0)	0.096	**-**	**-**	**-**
P-LCR, %	27.8 (26.0, 29.1)	28.3 (25.0, 34.9)	23.6 (17.7, 36.4)	0.412	–	–	–

Data are median (interquartile range) unless otherwise stated. WBC, white blood cell; RBC, red blood cell; HGB, hemoglobin; PLT, platelet; HCT, hematocrit; RDW-CV, coefficient of variation of red blood cell distribution width; RDW-SD, standard deviation of red blood cell distribution width; PDW, platelet distribution width; P-LCR, platelet-large cell ratio. Bold font indicates statistical significance.

**Table 2:** Clinical characteristics in SLE patients with and without PAH.

Variables	SLE-non-PAH (n=36)	SLE-PAH (n=18)	*p*-value
SLE duration, months (median, quartile)	48.0 (2.6, 117.0)	79.0 (11.8, 240.0)	0.053
SLEDAI-2K	12±7	10±8	0.381
Laboratory parameters			
CRP, mg/dl	0.26 (0.18, 1.35)	0.67 (0.40, 1.20)	0.066
C3, mg/dl	53.4±19.5	65.8±27.5	0.063
C4, mg/dl	13.5±6.0	15.2±6.4	0.051
Anti-dsDNA positive (n, %)	19 (52.8%)	7 (38.9%)	0.336
Anti-nRNP positive (n, %)	14 (38.9%)	13 (72.2%)	**0.021**
ALB, g/L	31.9±6.0	31.4±4.4	0.772
ALT, U/L	20 (13, 42)	17 (13,35)	0.890
Creatinine, μmol/L	54 (41, 61)	64 (46, 99)	0.189
Uric acid, μmol/L	309.8±113.9	413.8±160.9	**0.009**
PT, seconds	11.3 (10.7, 11.7)	11.8 (11.3, 25.1)	**0.007**
PT-INR (median, quartile)	1.03 (0.98, 1.07)	1.08 (1.03, 2.27)	**0.007**
APTT, seconds (median, quartile)	29.5 (27.3, 34.8)	33.0 (29.2, 37.6)	0.104
TT, seconds (median, quartile)	21.0 (19.4, 24.2)	20.2 (19.4, 22.1)	0.304
Fibrinogen, g/L (median, quartile)	2.56 (2.31, 2.95)	3.41 (2.45, 4.02)	**0.040**
D-Dimer, ng/mL (median, quartile)	742 (379, 2404)	735(482, 1208)	0.474
SLE manifestations (n, %)			
Nervous involvement	3 (8.3%)	1 (5.6%)	0.713
Renal involvement	21 (58.3%)	9 (50.0%)	0.561
Haematological involvement	29 (80.6%)	13 (72.2%)	0.487
Rash	11 (30.6%)	3 (16.7%)	0.177
Arthritis	5 (13.9%)	2 (11.1%)	0.775
Serositis	11 (30.6%)	12 (66.7%)	**0.011**
Raynaud phenomenon	1 (2.8%)	4 (22.2%)	**0.003**
Gastrointestinal involvement	7 (19.4%)	1 (5.6%)	0.176
UCG			
PASP, mmHg	22 (18, 26)	61 (50, 73)	**<0.001**
RV diameter, mm	35 (32, 37)	41 (37,49)	**0.008**
Pericardial effusion (n, %)	3 (8.6%)	7 (38.9%)	**0.008**
6MWD*, m (median, quartile)	–	365 (0, 414)	–
BNP, ng/L (median, quartile)	26.7 (14.8, 127.3)	390.0 (82.7, 912.5)	**0.003**
Medications			
Glucocorticoids (n, %)	22 (61.1%)	13 (72.2%)	0.420
Immunosuppressants (n, %)	13 (36.1%)	8 (44.4%)	0.554
Anticoagulant	1 (3.3%)	9 (50%)	**<0.001**

CRP, C-reactive protein; C3/4, Complement 3/4; anti-dsDNA, anti-double strand DNA antibody; anti-RNP, anti-nuclear ribonucleoprotein antibody; ALB, Albumin; ALT, Alanine transaminase; PT, prothrombin time; PT-INR, prothrombin time- International Normalized Ratio; APTT, activated partial thromboplastin time; TT, thrombin time; PASP, pulmonary artery systolic pressure; RV, right ventricle; *6MWD, 6-min walk distance, 0m was assigned to patients who were admitted to the hospital with symptoms of obvious right heart failure and were unable to tolerate the test; BNP, brain natriuretic peptide. Bold font indicates statistical significance.

### 3.2 Circulating EVs in SLE patients with and without PAH

**Figure 2:**
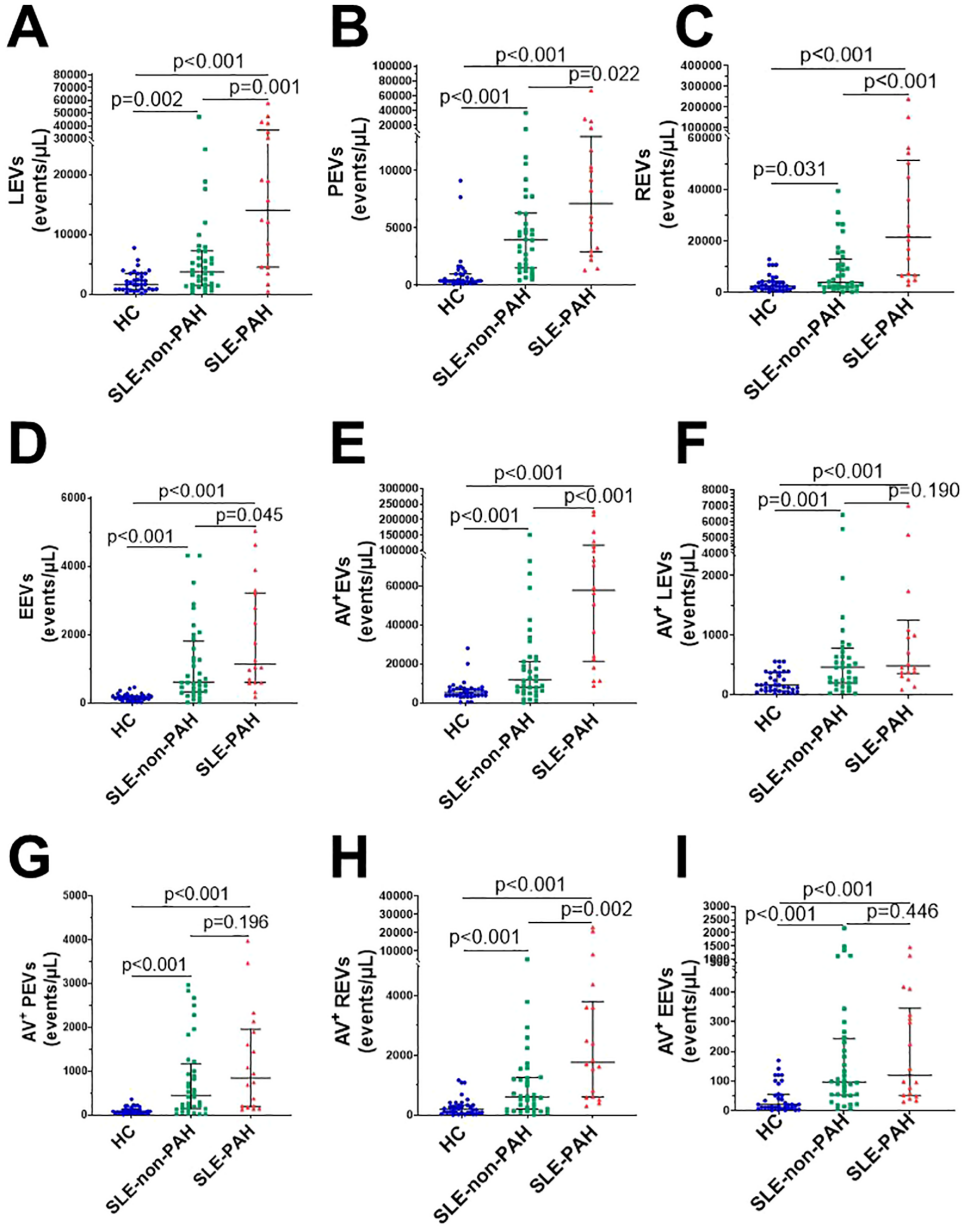
Circulating EVs in SLE patients with or without PAH, compared with healthy control groups. **(A)** Statistical results of LEVs; **(B)** PEVs; **(C)** REVs; **(D)** EEVs; **(E)** Annexin V^+^EVs; **(F)** Annexin V^+^LEVs; **(G)** Annexin V^+^ PEVs **(H)** Annexin V^+^ REVs; **(I)** Annexin V^+^EEVs in SLE patients with/without PAH, and HC group.

When we compared all circulating EVs among SLE patients with or without PAH, and healthy controls, we found significantly increased plasma levels of LEVs (CD45^+^LEVs), PEVs (CD41a^+^PEVs), REVs (CD235a^+^REVs), EEVs (CD144^+^EEVs), and Annexin V^+^EVs in SLE patients with or without PAH as compare to healthy controls (**Figures 2A–E**). In addition, the dual stainings of Annexin V^+^ LEVs, Annexin V^+^ PEVs, Annexin V^+^ REVs, and Annexin V^+^ EEVs were also elevated in both SLE patient groups as comparted to those in HC subjects (**Figures 2F–I**), indicating the increased generation of EVs from leukocytes, platelets, RBCs, and vascular endothelium in SLE patients no matter if they have PAH or not.

**Figure 3:**
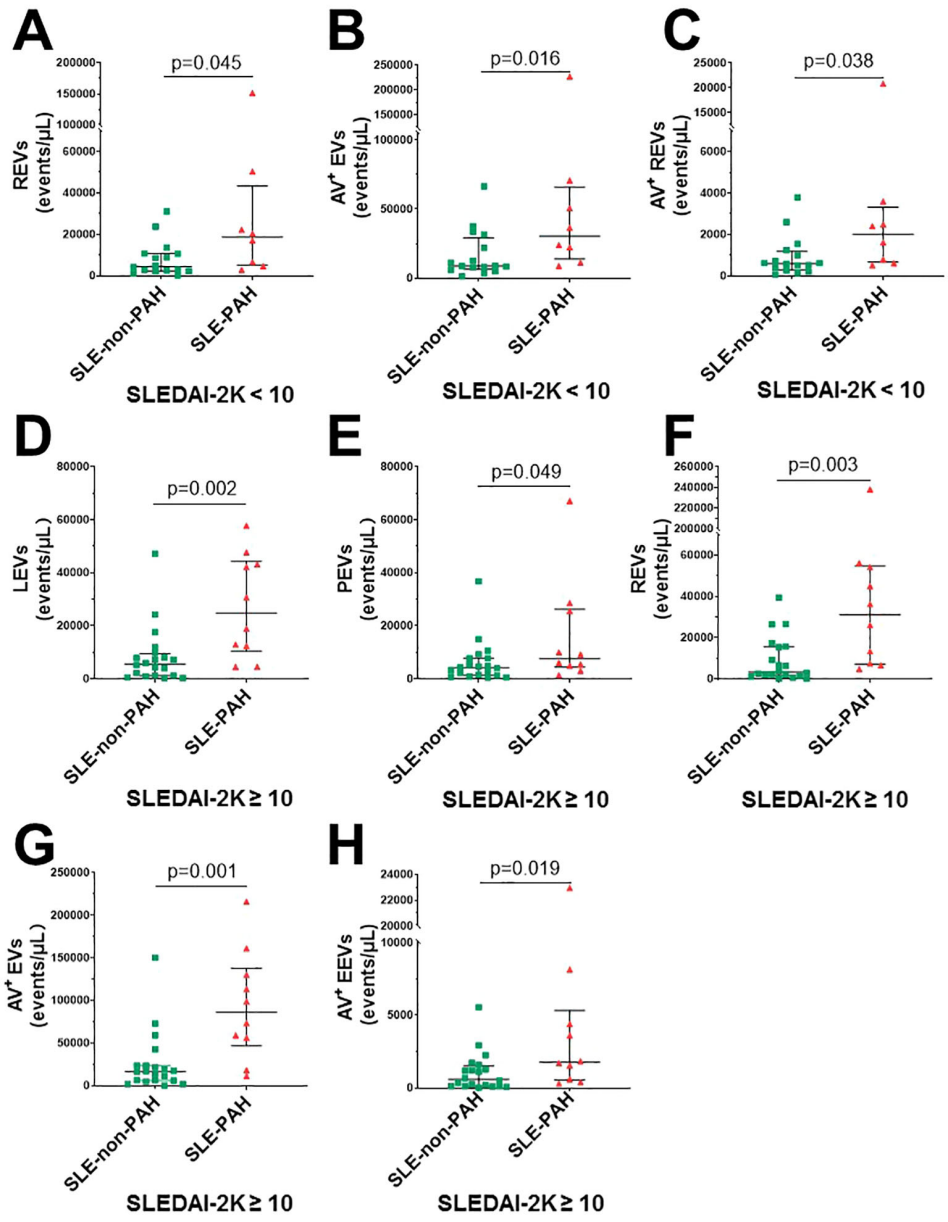
Circulating EVs in different SLE activity. **(A)** SLE-PAH patients (n=8) compared with SLE-non-PAH patients (n=16) statistical results of REVs; **(B)** Annexin V^+^ EVs; **(C)** Annexin V^+^REVs when SLEDAI-2K<10. **(D)** SLE-PAH patients (n=10) compared with SLE-non-PAH patients (n=20) statistical results of LEVs; **(E)** PEVs. **(F)** REVs; **(G)** Annexin V^+^EVs; **(H)** Annexin V^+^ REVs when SLEDAI-2K≥10.

To further explore the relationship EVs and PAH in SLE patients with or without PAH, we compared single-labeled EVs, i.e. LEVs, PEVs, REVs, EEVs, and EVs, as well as the dual-labeled EVs, i.e. Annexin V^+^ LEVs, Annexin V^+^ PEVs, Annexin V^+^ REVs, and Annexin V^+^ EEVs, between the SLE-PAH and SLE-non-PAH groups (**Table 3**). All single-labeled EVs (LEVs, PEVs, REVs, EEVs and Annexin V^+^ EVs), and the dual-labeled EVs (Annexin V^+^ REVs) were significantly increased in SLE-PAH group as comparted to those in SLE-non-PAH group (**Figure 2**), suggesting the potential role of circulating EVs in PAH. In order to eliminate the impact of SLE disease severity, patients were further divided into an inactive/mild SLE group (SLEDAI-2K<10) and a moderate/high SLE group (SLEDAI-2K≥10). Interestingly, the plasma levels of Annexin V^+^EVs, REVs, and Annexin V^+^ REVs were elevated in SLE-PAH patients with inactive/mild SLE activities; while the SLE-PAH patients with moderate/high SLE activities have increased plasma levels of LEVs, PEVs, REVs, Annexin V^+^EVs, and Annexin V^+^ REVs (**Table 4**; **Figure 3**).

**Table 3:** Circulating EVs and their cellular origin in different groups of subjects.

Origin	Healthy controls (n=36)	SLE-non-PAH (n=36)	SLE-PAH (n=18)	*p*-value		
				SLE-non-PAH vs. HC	SLE-PAH vs. HC	SLE-non-PAH vs. SLE-PAH
Total procoagulant						
Annexin V^+^ EVs	5263 (3778, 7481)	11959 (6029, 23610)	57783 (21319, 117546)	**<0.001**	**<0.001**	**<0.001**
Leukocytes						
LEVs	1595 (793, 3457)	3697 (1450, 7216)	14285 (4512, 36833)	**0.002**	**<0.001**	**0.001**
Annexin V^+^ LEVs	158 (68, 370)	459 (198, 777)	483 (355, 1250)	**0.001**	**<0.001**	0.190
Platelets						
PEVs	401 (297, 997)	3965 (1531, 6296)	7129 (2920, 12965)	**<0.001**	**<0.001**	**0.022**
Annexin V^+^ PEVs	84 (55,134)	453 (152, 1169)	848 (197, 1963)	**<0.001**	**<0.001**	0.196
Red blood cells						
REVs	2395 (1187, 4164)	3909 (2027, 12900)	21365 (6643, 51282)	**0.031**	**<0.001**	**<0.001**
Annexin V^+^ REVs	187 (70, 330)	598 (196, 1262)	1769 (608, 3804)	**<0.001**	**<0.001**	**0.002**
Endothelial cells						
EEVs	163 (109, 203)	615 (327, 1808)	1143 (604, 3219)	**<0.001**	**<0.001**	**0.045**
Annexin V^+^ EEVs	20 (10, 55)	97 (52, 242)	120 (52, 346)	**<0.001**	**<0.001**	0.446

Bold font indicates statistical significance.

**Table 4:** Circulating EVs in SLE patients with different severity.

Origin	SLEDAI-2K<10		*p*-value	SLEDAI-2K≥10		*p*-value
	SLE-non-PAH (n=16)	SLE-PAH(n=8)		SLE-non-PAH (n=20)	SLE-PAH(n=10)	
Total procoagulant						
Annexin V^+^	8887 (6420, 29197)	30323 (14045, 65717)	**0.016**	16493 (5925, 23610)	86113 (46839, 137743)	**0.001**
Leukocytes						
LEVs	3067 (1580, 5051)	9371 (2084, 18232)	0.093	5574 (1204, 9416)	24803 (10415, 44269)	**0.002**
Annexin V^+^ LEVs	421 (229, 780)	477 (193, 1481)	0.610	489 (142, 777)	711 (355, 1672)	0.183
Platelets						
PEVs	3664 (1605, 5892)	5755 (2347, 11406)	0.264	4161 (1249, 7746)	7597 (4429, 26261)	**0.049**
Annexin V^+^ PEVs	412 (108, 1152)	622 (155, 2021)	0.383	453 (202, 1199)	1098 (208, 1963)	0.286
Red blood cells						
REVs	4514 (2382, 10724)	18694 (5168, 43325)	**0.045**	3336 (1510, 15559)	31062 (7201, 54661)	**0.003**
Annexin V^+^ REVs	598 (291, 1178)	2008 (660, 3319)	**0.038**	590 (119, 1497)	1769 (541, 5322)	**0.019**
Endothelial cells						
EEVs	536 (327, 825)	1003 (390, 2734)	0.214	1174 (270, 2221)	2049 (669, 3368)	0.155
Annexin V^+^ EEVs	71 (35, 163)	96 (63, 268)	0.192	126 (64, 549)	261 (45, 413)	0.948

Bold font indicates statistical significance.

### 3.3 Correlation between circulating EVs and severity of PAH

To explore the relationship between EVs and PAH, we analyzed the correlation analysis between each EVs subgroup and UCG parameters, as only 7 SLE-PAH patients had RHC results at the time of sample collection. The results showed that LEVs, REVs, EEVs, and Annexin V^+^EVs, were positively correlated with PASP in the combined groups of all SLE patients (n=54). Further analysis of SLE-PAH patients showed positive correlation between EEVs and PASP (r=0.274, p=0.045). In addition, plasma levels of PEVs and EEVs were positively correlated with RVD. These results demonstrated significant relationship between platelets and endothelial cell-derived EVs and PAH severity (**Table 5**).

**Table 5:** Correlation between EVs subgroups and UCG parameters in all SLE or SLE-PAH patients.

MPs subgroups	SLE (n=54)		SLE-PAH (n=18)			
	PASP		PASP		RVD	
	r	*p*-value	r	*p*-value	r	*p*-value
Total						
Annexin V^+^	0.361	**0.007**	0.270	0.278	0.268	0.282
Leukocytes						
LEVs	0.293	**0.032**	0.409	0.092	0.405	0.096
Annexin V^+^ LEVs	0.076	0.585	0.268	0.282	0.184	0.464
Platelets						
PEVs	0.196	0.156	0.226	0.366	0.501	**0.034**
Annexin V^+^ PEVs	0.081	0.561	0.350	0.155	0.294	0.237
Red blood cells						
REVs	0.274	**0.045**	0.257	0.304	0.455	0.058
Annexin V^+^ REVs	0.238	0.082	0.149	0.555	0.302	0.223
Endothelial cells						
EEVs	0.274	**0.045**	0.506	**0.032**	0.508	**0.031**
Annexin V^+^ EEVs	0.085	0.540	0.429	0.076	0.240	0.337

PASP, pulmonary arterial systolic pressure; RVD, right ventricular diameter. Bold font indicates statistical significance.

### 3.4 Receiver operating characteristic analysis of circulating EVs in SLE patients with PAH

**Figure 4:**
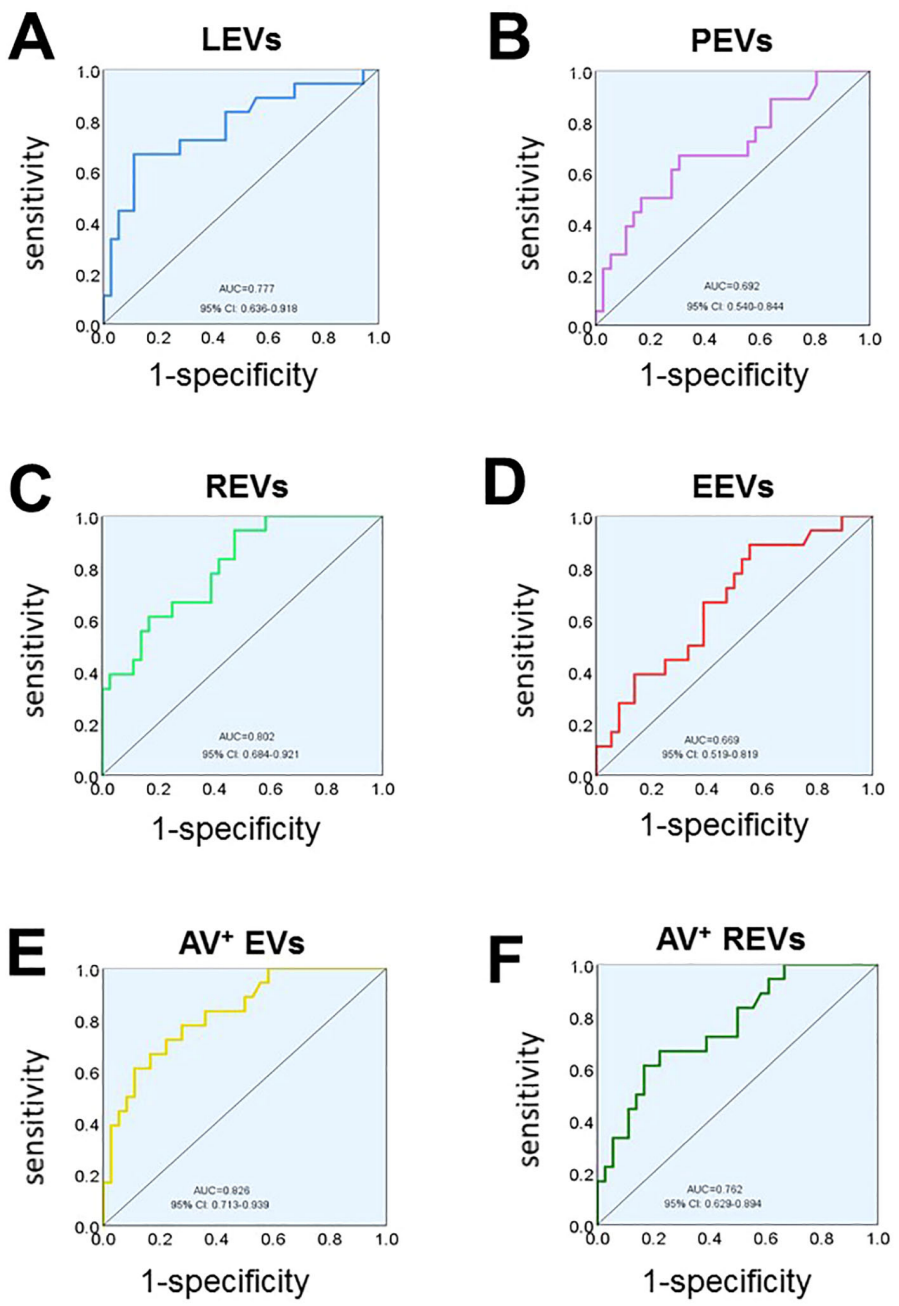
Receiver operating characteristic (ROC) analysis. ROC analysis shows the ability of LEVs **(A)**, PEVs **(B)**, REVs **(C)**, EEVs **(D)**, Annexin V^+^EVs **(E)**, Annexin V^+^ REVs **(F)** concentrations in discriminating SLE-PAH (n=18) from SLE-non-PAH (n=36).

To examine the specificity and sensitivity of circulating EVs, the ROC analyses found that plasma levels of LEVs, PEVs, REVs, EEVs, Annexin V^+^EVs, and the dual-labeled Annexin V^+^ REVs could clearly distinguish SLE-PAH from SLE-non-PAH (**Figures 4A–F**). Among the characteristics of circulating EVs, Annexin V^+^ EVs was the best predictor of PAH in patients with SLE for both specificity and sensitivity (**Figure 4E**; **Table 6**), suggesting the importance of Annexin V-positive, PS-exposing EVs in the development of PAH in SLE patients. In addition, LEVs are significant predictors of SLE-PAH (**Table 6**), suggesting that leukocyte-derived LEVs and their associated cago molecules play an important role in SLE-PAH development. Importantly, both REVs and Annexin V^+^ REVs predict SLE-PAH (**Table 6**), implicating RMV-associated PS in SLE-PAH pathogenesis. On the other hand, EEVs also significantly predict SLE-PAH (**Table 6**), indicating the involvement of endothelial damage in SLE-PAH pathogenesis.

**Table 6:** Circulating EVs concentrations for distinguish SLE-PAH from SLE-non-PAH.

EVs types	Concentrations (events/uL)	Area	*p*-value	Sensitivity	Specificity	95% CI
Total procoagulant						
Annexin V^+^	46698	0.826	**<0.001**	0.611	0.889	0.713~0.939
Leukocytes						
LEVs	12023	0.777	**0.001**	0.667	0.889	0.636~0.918
Annexin V^+^ LEVs	297	0.610	0.190	0.833	0.444	0.452~0.768
Platelets						
PEVs	4882	0.692	**0.022**	0.667	0.694	0.540~0.844
Annexin V^+^ PEVs	669	0.609	0.196	0.611	0.611	0.449~0.768
Red blood cells						
REVs	4388	0.802	**<0.001**	0.944	0.528	0.684~0.921
Annexin V^+^ REVs	1597	0.762	**0.002**	0.611	0.833	0.629~0.894
Endothelial cells						
EEVs	569	0.669	**0.045**	0.889	0.444	0.519~0.819
Annexin V^+^ EEVs	282	0.564	0.446	1.000	0.194	0.391~0.716

Bold font indicates statistical significance.

### 3.5 Associations between circulating EVs and PAH in SLE

To determine the prediction ability of circulating EVs and other associated clinical and laboratory indicators (based on variables in **Tables 1**, **2** and clinical implication) on PAH in SLE patients, we conducted univariate and multivariate logistic regression analyses (**Table 7**). Age, sex, and SLE disease activity were not included in the conditional logistic regression model as the groups were matched for them, and UCG parameters were not included because of their role in the inclusion and exclusion criteria for PAH. Anticoagulant was excluded because it was the treatment strategy based on a comprehensive assessment of the patient’s condition, and it was unrelated to the differential diagnoses. In univariate logistic regression analyses, the levels of LEVs (>12023 events/μL), PEVs (>4882 events/μL), REVs (>4388 events/μL), EEVs (>569 events/μL), Annexin V^+^EVs (>46698 events/μL), and Annexin V^+^REVs (>1597 events/μL), as well as RBC-SD, RBC-CV, UA, positive anti-nRNP antibody and serositis were risk factors of PAH. Most importantly, LEVs and REVs, serositis, and positive anti-nRNP antibody were independently associated with the development of PAH in SLE patients (**Table 7**), indicating that LEVs and REVs play an important role in development of PAH in SLE patients.

**Table 7:** Univariate and multivariate Logistic regression analysis for identifying PAH in SLE patients (n=54).

Variables	Univariate analysis		Multivariate analysis	
	Odds ratio (95% CI)	*p*-value	Odds ratio (95% CI)	*p*-value
LEVs >12023 events/μL	16.000 (3.834-66.763)	**<0.001**	7.376 (1.067-50.988)	**0.043**
PEVs >4882 events/μL	4.545 (1.356-15.238)	**0.014**	–	
REVs >4388 events/μL	19.000 (2.280-158.336)	**0.006**	31.297 (2.157-454.004)	**0.012**
EEVs >569 events/μL	6.400 (1.279-32.027)	**0.024**	–	
Annexin V^+^ EVs >46698 events/μL	12.571 (3.080-51.315)	**<0.001**	–	
Annexin V^+^ REVs >1597 events/μL	7.857 (2.161-28.568)	**0.002**	–	
RBC-SD	1.122 (1.033-1.218)	**0.006**	–	
RBC-CV	1.267 (1.018-1.576)	**0.034**	–	
Uric acid	1.006 (1.001-1.012)	**0.019**	–	
anti-nRNP positive	4.086 (1.194-13.978)	**0.025**	10.278 (1.365-77.381)	**0.024**
serositis	4.545 (1.356-15.238)	**0.014**	10.186 (1.375-75.473)	**0.023**

RDW-CV, coefficient of variation of red blood cell distribution width; RDW-SD, standard deviation of red blood cell distribution width; anti-RNP, anti-nuclear ribonucleoprotein antibody. Bold font indicates statistical significance.

## 4 Discussion

Circulating EVs have been reported to be associated with disease severity in SLE patients (24). In PAH, studies have investigated the differences of specific subsets of EVs in patients with iPAH, hPAH, and CTD-PAH (25, 26). However, no studies have investigated the circulating EVs in SLE-PAH patients. Here, we showed elevated circulating EVs, including LEVs, PEVs, REVs, EEVs, Annexin V^+^EVs, and double-labeled Annexin V^+^REVs, in SLE-PAH patients compared to SLE-non-PAH patients. The results suggest that these circulating EV subgroups are related to the pathologic conditions of PAH in patients with SLE.

As discussed above, studies from our the other groups have shown that Annexin V^+^EVs have procoagulant phospholipid activities due to their exposure of PS (27). In this study, we demonstrated that total Annexin V^+^EVs and Annexin V^+^ EVs from different circulating cells or endothelial cells were significantly increased in both SLE-non-PAH and SLE-PAH patients compared to HCs, indicating the involvement of EVs, particularly the EVs with PS exposure, in the development of SLE. Importantly, the levels of Annexin V^+^EVs in SLE-PAH patients were significantly higher than in SLE-non-PAH patients, suggesting the importance of procoagulant EVs in the development of PAH in SLE patients. Given the established role of Annexin V-positive EVs with PS exposure in procoagulation (27), these results support the pathologic involvement of EVs and the key role of a pro-thrombotic condition in the development of PAH in SLE.

RBCs are the most abundant circulating blood cells (28), and the primary contributors to the circulating EV population (29). In our study, we demonstrated that REVs emerged as the primary source of PS-exposing EVs among all cell types investigated, highlighting their potential role in the prothrombotic conditions contributing to PAH development in SLE patients. RBCs are known to release REVs in response to various stimuli, such as shear stress, complement activation, pro-apoptotic stimulation, and cellular injury (30). Previous studies have demonstrated that altered shear stress in PAH patients (31) is associated with abnormal erythrocyte morphology (32). RBC with increased RDW often exhibit abnormal morphology. In sickle cell disease, a condition characterized by increased RDW, both REV release and PS exposure on erythrocytes are increased, accompanied by elevated coagulation activation markers (33). The number of REVs was negatively correlated with clotting time, regardless of PS exposure (34). In addition to the effects of REV-associated PS, REVs also sustainably consume anticoagulant protein C and protein S, contributing to prothrombotic conditions (35). Furthermore, REVs derived from aged erythrocytes can induce pulmonary microthrombi through p-selectin in mice (36). Moreover, REVs were one of the most abundant EV subtypes in both blood and atherosclerotic plaques of patients with coronary heart diseases (37). Studies also found that REVs exert a more pronounced influence on coagulation than PEVs (38, 39). Collectively, these studies suggest that REVs exert significant procoagulant effects through different mechanisms. Hypercoagulability and even thrombosis are widely recognized as key pathogenic mechanisms in iPAH (40). While some SLE-PAH patients with hypercoagulability have been reported (5), the efficacy of anticoagulant therapy in improving survival remain controversial. Our study revealed higher levels of Annexin V^+^REVs in SLE-PAH compared with SLE-non-PAH, accompanied by increased RDW-SD and RDW-CV. These findings suggest a potential role of RBCs and their procoagulant REVs in the pathogenesis of SLE-PAH. However, coagulation times were not shortened in the SLE-PAH group; in fact and PT and PT-INR were prolonged. This paradoxical finding may be attributed to the fact that SLE-PAH patients were more likely to receive anticoagulant therapy (9 of 18 in SLE-PAH *vs.* 1 of 36 in SLE-non-PAH patients) due to their more severe overall clinical condition.

It’s known that activation of leukocytes and platelets is enhanced in PAH patients (41–43). Importantly, our study demonstrated increased levels of LEVs and PEVs in SLE-PAH patients. These results are consistent with previous research in PAH (25). Furthermore, PEVs can mediate leukocyte-platelet interaction, triggering activation of leukocytes and subsequent release of LEVs (44). In addition to the procoagulant properties of MV-associated PS (13, 14), previous studies have shown that LEVs and PEVs can induce endothelial injury, release of EEVs and inflammatory factors, proliferation of vascular smooth muscle cells, and intimal thickening (45–48). As circulating EEVs result from activated or apoptotic vascular ECs or damaged vascular endothelium, circulating EEVs could serve as a biomarker of vascular injury, indicating the pathologic conditions with impaired vasculature in patients (30). Many sentinel events, including those triggered by LEVs and PEVs, could induce endothelial injury, increasing the release of EEVs and worsening endothelial dysfunction. Since CD144 is a constituent marker of ECs, highly increased levels of CD144^+^EEVs in circulation indicate impairment of vascular endothelium and their contribution to PAH development in SLE-PAH patients. Furthermore, Amabile reported a positive correlation between blood levels of CD144^+^EEVs and hemodynamic severity of PAH (26). In line, we also found that the elevated levels of EEVs in patients with SLE-PAH were positively correlated with PASP and RVD, suggesting that circulating EEV levels may reflect the severity of SLE-PAH. However, no correlation between EEV levels and RHC parameters was found. Interestingly, our results indicate that there was a close relationship between PEVs, EEVs, and LEVs, and their interactions synergistically participating the SLE-PAH development. There were good correlations between these three subgroups of EVs which was consistent with previous studies (25). All the above indicates that systemic inflammation, endothelial injury, and hypercoagulation state co-exist simultaneously and interact each other during PAH development in SLE-PAH. Although no distinction was found in conventional blood cell counts, such as WBC, RBC, and platelets, between SLE-non-PAH and SLE-PAH patients, the levels of LEVs, REVs, and PEVs were significantly elevated in SLE-PAH patients, indicating that circulating EVs, rather than their parental cells, may be more indicative of the underlying pathological processes in SLE-PAH. Furthermore, statistical analysis also showed no relationship between the levels of circulating EVs and the numbers of their corresponding parental cells in SLE patients with or without PAH (P >0.05, data not shown).

A recent study found that SLE-PAH could be classified into vasculitic and vasculopathic subtypes based on clinical characteristics in patients (49). SLEDAI>9 was an independent predictor for the high-risk vasculitic subtype SLE-PAH (49). The vasculitic subtype of SLE-PAH patients, with more intensive systemic inflammatory responses and more severe systemic symptoms, are more likely to respond to intensive immunosuppressive therapy, making the PAH process reversible in these patients (50). In contrast, the vasculopathic subtype, however, tends to be non-inflammatory vascular remodeling during its PAH pathogenesis. The above classifications are consistent with the results of the present study. Accordingly, the subgroups were divided according to SLEDAI-2K in our study. When the disease activity was low (SLEDAI-2K<10), levels of circulating EEVs, PEVs, and LEVs in SLE-PAH patients showed no difference compared to SLE patients without PAH, while their Annexin V^+^EVs increased significantly. This suggested that the inflammatory responses were not intensive although their hypercoagulability was more pronounced when SLE was not severe. In addition, their levels of REVs also increased, suggesting that the role of erythrocytes and the REVs in PAH pathogenesis under these conditions which deserves more research in the future. In contrast, the levels of LEVs, PEVs, REVs, Annexin V^+^EVs, and Annexin V^+^REVs were increased in the SLE patients with SLEDAI-2K≥10. These findings suggest that this patient group exhibited characteristics of vasculitic PAH, marked by not only hypercoagulation state, but also hyperinflammatory state with more pronounced activation of leukocytes, platelets, and erythrocytes *in vivo* in their bloodstream.

PASP is the most important indicator in UCG for screening and assessment of PAH. In our study, circulating LEVs, REVs, EEVs, and AV^+^EVs were positively correlated with PASP in all SLE patients, suggesting the potential contributions of these circulating EVs to the development of PAH in SLE patients. Importantly, circulating EEVs were positively correlated with PASP in SLE-PAH patients, suggesting the value of EEVs in assessment of PAH severity in patients with SLE-PAH. As the most intuitive indicator of right ventricular dilation, the increased RVD was considered to be a poor and indirect prognostic marker in PAH patients (51). Our results showed that PEVs and EEVs were positively correlated with RVD in patients with SLE-PAH, indicating that platelet- and endothelial-derived EVs are also valuable in assessing the severity of right heart dysfunction, which may however be the consequences of the increased PASP in these patients.

To further evaluate the usefulness of circulating EVs measured in our study, ROC curve analysis indicated that all single-labeled EVs and dual-labeled Annexin V^+^REVs are valuable to serve as potential biomarkers to identify PAH in SLE patients. Annexin V^+^ EVs was the best predictor of PAH in patients with SLE for both specificity and sensitivity, indicating the importance of PS-exposing EVs in PAH development. In addition, circulating LEVs were significant predictors of SLE-PAH, suggesting the importance of inflammatory leukocytes and their released LEVs in pathogenesis of SLE-PAH. Furthermore, logistic regression analysis indicated that circulating LEVs, REVs, and anti-NRNP antibody, as well as serositis were independent predictors of PAH in SLE. Among these variables, anti-nRNP antibody and serositis have been widely recognized as predictors of PAH (52, 53). Most importantly, our work demonstrated that levels of LEVs and REVs could also be independent biomarkers of SLE-PAH. The ability of EVs to identify PAH in patients with SLE has not yet been previously reported.

This study however has some limitations. First, owing to the fact of rare incidence of PAH in SLE, the number of SLE-PAH patients recruited to this study was limited. However, we strictly matched these subjects with SLE patients without PAH and healthy controls, which could partially eliminate bias. Second, only seven SLE-PAH patients had RHC results at the time of blood sample collection. The criteria for SLE-PAH patients included in this study were RHC compliance or PASP > 50 mmHg, as indicated by UCG according to the previous studies (54, 55). Among them, 11 patients were not tested for RHC owing to factors such as disease intolerance or patient willingness. However, the UCG results and comprehensive clinical evaluation of these patients were highly indicative of PAH. Peak tricuspid regurgitation velocitys of all these 11 patients exceed 3.4m/s, which indicated high echocardiographic probability of PAH (23). Then each patient was also assessed for probability of PAH with echocardiographic signs according to the 2015 European Society of Cardiology (ESC) and the European Respiratory Society (ERS) guidelines (**Supplementary Table 1**) (23). All these patients had clinical presentations of PAH, such as shortness of breath after exertion, fatigue, syncope, abdominal distension, dry cough, lower limb oedema, and so on. Auscultation of the second heart sound for these patients showed P2>A2. Importantly, UCG of multiple follow-up visits for these patients persistently indicated PAH, which was not transient or reversible. So, we enrolled these 11 patients in this study who met the clinical diagnostic criteria for PAH. Owing to the limited numbers of RHC results, we explored the relationship between PAH-associated UCG parameters and EV subgroups in the correlation analysis to compensate for the deficiency of incomplete RHC data to some extent. In addition, studies on the mechanisms of EVs in various subgroups, especially REV, in SLE-PAH are ongoing, and we will conduct follow-up reports.

## 5 Conclusion

This study demonstrated that all single-labeled LEVs, PEVs, REVs, EEVs and Annexin V^+^ EVs, as well as dual-labeled Annexin V^+^ REVs were significantly increased in SLE-PAH patients compared to SLE-non-PAH patients. In patients with inactive/mild SLE, the procoagulant activity of EVs increased in PAH patients. In patients with moderate/high SLE, both procoagulant activity and hyperinflammatory state mediated by EVs were evident in patients with PAH. Therefore, our study indicated that specific subgroups of EVs are involved in the development of PAH in SLE patients. In addition to the widely reported anti-nRNP antibody and serositis, our study demonstrated that circulating LEVs and REVs are independent biomarkers of SLE-PAH. This work indicated the clinical usefulness of EVs in identification, diagnosis, and management of PAH in patients with SLE.

## Data Availability

The original contributions presented in the study are included in the article/**Supplementary Material**. Further inquiries can be directed to the corresponding authors.
