# Supplementary material for: Circulating extracellular vesicles as novel biomarkers for pulmonary arterial hypertension in patients with systemic lupus erythematosus
Source: Front Immunol. 2024 Sep 19;15:1374100. doi: 10.3389/fimmu.2024.1374100 (PMC11446868; doi:10.3389/fimmu.2024.1374100)
Supplement: Supplementary file 1 [file Table1.pdf]

Supplement Table 1 Echocardiographic signs suggesting PAH for the 11 SLE-PAH patients without RHC.

Patients	Peak tricuspid regurgitation velocity (m/s)	PA systolic pressure (mmHg)	PA diameter (mm)	Early diastolic pulmonary regurgitation velocity (m/s)	Right ventricle/left ventricle basal diameter ratio	Right cardiac enlargement
1	432	58	26	2.95	1.02	Yes
2	452	92	36	2.62	1.79	Yes
3	368	52	29	2.71	1.19	Yes
4	403	70	30	2.69	1.14	Yes
5	379	57	26	2.85	1.42	Yes
6	497	90	31	2.73	1.08	Yes
7	431	51	32	2.63	1.36	Yes
8	365	63	30	2.61	1.34	Yes
9	387	52	31	2.67	1.37	Yes
10	423	61	29	2.77	1.13	Yes
11	372	65	33	2.48	1.10	Yes

PA: pulmonary artery
